# Spondylodiscitis Following Oxygen–Ozone Therapy: A Case Report of *Lactobacillus iners* Infection and a Systematic Literature Review

**DOI:** 10.3390/diseases14030115

**Published:** 2026-03-23

**Authors:** Calogero Velluto, Giovan Giuseppe Mazzella, Michele Inverso, Maria Ilaria Borruto, Andrea Perna, Riccardo Totti, Laura Scaramuzzo, Luca Proietti

**Affiliations:** 1 Department of Aging, Orthopaedic and Rheumatological Sciences, Fondazione Policlinico Universitario Agostino Gemelli IRCCS, 00136 Rome, Italy; calogero.velluto@guest.policlinicogemelli.it (C.V.); ggmazzella@gmail.com (G.G.M.); inversomichele7@gmail.com (M.I.); maria.ilaria.borruto@gmail.com (M.I.B.); totti.riky@gmail.com (R.T.); luca.proietti@policlinicogemelli.it (L.P.); 2 Department of Orthopaedics and Traumatology, Fondazione Casa Sollievo della Sofferenza IRCCS, 71013 San Giovanni Rotondo, Italy; perna.andrea90@gmail.com

**Keywords:** spine infection, ozone therapy, spondylodiscitis, systematic review, *Lactobacillus iners*

## Abstract

Background: Oxygen–ozone (O_2_–O_3_) therapy is a minimally invasive treatment for discogenic lumbar pain. Although rare, spinal infections—specifically spondylodiscitis—have been reported following intradiscal injections. To date, *Lactobacillus iners* has not been described as a causative agent in this context. Case Presentation: A 55-year-old immunocompetent woman presented with progressive lumbosciatica and elevated inflammatory markers three months after intradiscal O_2_–O_3_ therapy. MRI revealed L4–L5 spondylodiscitis with paravertebral involvement. Surgical biopsy confirmed *L. iners* as the pathogen. She underwent decompression and received targeted intravenous antibiotics, achieving full clinical and radiological recovery. Methods: A systematic literature review was performed using PubMed, MEDLINE, and Scopus to identify reports of spondylodiscitis following oxygen–ozone therapy. Six cases were included based on predefined inclusion criteria. Results: The 8 identified cases involved a range of pathogens, including *Staphylococcus aureus*, *Streptococcus beta-haemolyticus*, *Escherichia coli*, *Achromobacter xylosoxidans*, *Mycobacterium abscessus*, and *Streptococcus intermedius*, and one culture-negative infection. Clinical presentations varied from radiculopathy to sepsis. Management strategies encompassed both conservative (antibiotics alone) and surgical approaches, depending on neurological status and abscess formation. Outcomes were favorable in all cases except one fatality. Conclusions: This report is the first to describe *L. iners* spondylodiscitis in an immunocompetent patient following O_2_–O_3_ therapy. Clinicians should vigilantly evaluate post-infiltration spinal infections, maintain a low threshold for imaging and biopsy, and implement pathogen-targeted antibiotic regimens, with surgical intervention as needed.

## 1. Introduction

Spondylodiscitis (SD) and vertebral osteomyelitis (VO) are severe spinal infections associated with significant morbidity and potential neurological complications that require accurate diagnostic procedures, often involving prolonged antibiotic therapy, and, in some cases, surgical intervention [1].

The etiology of SD and VO is heterogeneous. It includes a broad spectrum of pathogens, although these infections are most commonly caused by pyogenic bacteria, atypical microorganisms, mycobacteria, fungi, and, in rare cases, viruses have also been reported [2].

According to microbiological data, *Staphylococcus* spp. accounted for 40.3% of SD cases, Mycobacterium tuberculosis accounted for 30.9% of cases, and other bacteria accounted for 28.3% of cases. Fungal (0.5%) and viral (0.1%) etiologies were identified only in a minority of cases [3].

Intradiscal O_2_–O_3_ therapy is a minimally invasive and relatively low-cost procedure that can be performed under CT or fluoroscopic guidance. The therapeutic principle relies on reducing disc volume and alleviating pressure on the adjacent nerve root, without compromising the spinal canal [4]. The diffusion of ozone into the surrounding tissues exerts direct anti-inflammatory and analgesic effects by inhibiting the synthesis of pro-inflammatory mediators, such as prostaglandins and bradykinins [5].

Despite its generally favorable safety profile, rare but severe complications have been reported following percutaneous spinal injection procedures, including spondylodiscitis and vertebral osteomyelitis. In these cases, pathogenesis is thought to differ from hematogenous spread and may involve direct inoculation during the procedure. However, in most published reports, the relationship between O_2_–O_3_ therapy and subsequent infection remains temporal and circumstantial, and a definitive causal link cannot be firmly established.

However, only a few cases are documented in the literature about the correlation between this procedure and SD or VO. To date, Lactobacillus iners has not been reported as a causative agent in this setting. Although *Lactobacillus* species are generally considered commensal microorganisms, emerging evidence suggests that certain strains may exhibit opportunistic behavior under specific conditions.

We reported the first case of spondylodiscitis caused by Lactobacillus iners, following oxygen–ozone therapy intradiscal injection performed for lumbar disc herniation. Although a direct causal relationship cannot be proven, the temporal sequence and the absence of alternative sources of infection suggest a plausible association.

The aim of this report is to describe this rare case of Lactobacillus iners spondylodiscitis, along with a systematic literature review of SD and VO cases following an oxygen therapy treatment for spine pathologies. By integrating clinical, microbiological, and therapeutic data, we aim to highlight diagnostic challenges, pathogen heterogeneity, and implications for the prevention and management of iatrogenic spinal infections.

## 2. Materials and Methods

### 2.1. Search Strategy

This study was conducted in accordance with the Preferred Reporting Items for Systematic Reviews and Meta-Analyses (PRISMA) guidelines [6] (Table S1). The protocol is registered on the International Prospective Register of Systematic Reviews (CRD420261306933).

A systematic literature search was independently performed by two authors (C.V. and G.G.M.) using PubMed, MEDLINE, and Scopus databases. The search strategy combined terms related to spinal infection, including “spondylodiscitis”, “vertebral osteomyelitis”, and “spinal infection”, with terms related to ozone treatment, such as “oxygen–ozone”, “ozone therapy”, and “intradiscal ozone”. When applicable, corresponding Medical Subject Headings (MeSH) terms were also included. Boolean operators “AND” and “OR” were used to combine search terms.

The search covered publications from January 2000 to May 2025. No restrictions were applied regarding study design. Only full-text articles published in English were considered eligible for inclusion. In addition, the reference lists of all included studies and relevant reviews were manually screened to identify further potentially eligible publications.

All retrieved records were imported into reference management software, and duplicate entries were removed before the screening process.

### 2.2. Inclusion and Exclusion Criteria

Studies were considered eligible if they reported cases of spondylodiscitis or vertebral osteomyelitis occurring after oxygen–ozone (O_2_–O_3_) therapy performed for spine-related disorders. Eligible study designs included case reports, case series, and retrospective or prospective observational studies.

To be included, articles were required to provide sufficient clinical detail to allow adequate case characterization, including at least patient demographics, clinical presentation, imaging findings, microbiological results, treatment strategy, and clinical outcome. In addition, only studies describing a clear temporal relationship between O_2_–O_3_ therapy and the onset of infection were considered.

Only full-text articles published in English were included.

Studies were excluded if they did not specify a temporal association between oxygen–ozone therapy and spinal infection; if they lacked essential clinical or microbiological information; or if alternative sources of infection could not be reasonably excluded. Reviews, editorials, expert opinions, conference abstracts, animal or cadaveric studies, and duplicated publications were also excluded.

**Figure 1:**
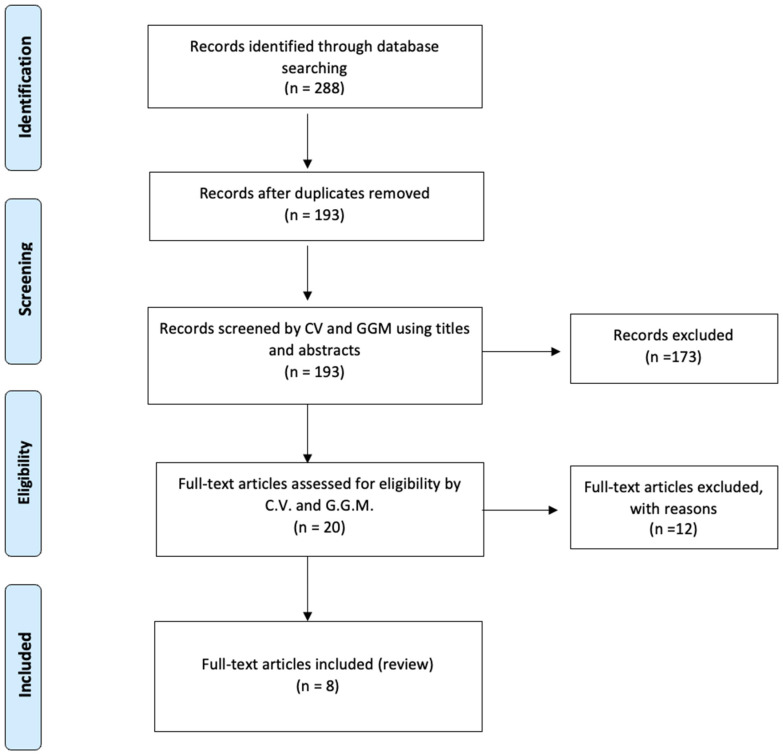
PRISMA flow-chart.

During the selection process, duplicate records were removed, and potentially overlapping cases were carefully assessed to avoid data redundancy. The study selection process is summarized in the PRISMA flow diagram (Figure 1).

### 2.3. Data Collection

Two authors (C.V. and G.G.M.) independently screened titles and abstracts for relevance to cases of spondylodiscitis following oxygen–ozone (O_2_–O_3_) therapy. Full texts of potentially eligible studies were then reviewed to verify compliance with the predefined inclusion and exclusion criteria.

Disagreements were resolved through discussion and consensus, with the involvement of a third reviewer (L.S.) when necessary.

For each included study, data were systematically extracted using a standardized data collection form. Extracted data included: patient demographics, indication for O_2_–O_3_ treatment, latency period between injection and symptom onset, clinical presentation, laboratory findings, microbiological findings, imaging modalities used for diagnosis, treatment strategies (antibiotic therapy, surgical intervention), duration of therapy, and clinical outcomes.

To ensure accuracy and consistency, all extracted data were cross-checked by at least two investigators against the original publications. When discrepancies or incomplete information were identified, the articles were re-evaluated and clarified through consensus.

### 2.4. Statistical Analysis

Data extraction and organization were conducted using Microsoft Excel (Microsoft Corporation, Redmond, WA, USA) to facilitate descriptive analyses.

Categorical variables were expressed as absolute numbers and corresponding percentages, whereas continuous variables were presented as means and standard deviations or medians and ranges, when appropriate, depending on data distribution and availability. Given the limited number of included studies and the predominance of case reports and small case series, no formal statistical comparisons were made. Due to the substantial clinical and methodological heterogeneity among the included studies, a quantitative meta-analysis was not considered appropriate.

Instead, a narrative synthesis supported by descriptive and summary tables was utilized to highlight the main findings and allow for qualitative comparisons between reported cases.

## 3. Case Report

We report the case of a 55-year-old female with no significant past medical history who presented with left-sided lumbosciatica beginning in January 2022. A lumbar spine MRI performed at that time revealed an L4–L5 disc herniation.

In March 2022, the patient underwent five sessions of oxygen–ozone (O_2_–O_3_) intradiscal therapy, which initially resulted in partial symptom relief. No immediate complications were reported following the procedure.

However, starting in July 2022, she experienced a progressive worsening of both low back and sciatic pain over a three-month period. Symptoms progressively increased in intensity and became refractory to conservative treatment.

In September 2022, routine blood tests revealed neutrophilic leukocytosis and an elevated C-reactive protein (CRP) level of 91 mg/L. Following her general practitioner’s advice, she began empirical antibiotic therapy with ciprofloxacin 500 mg twice daily for ten days (20 September 2022 to 30 September 2022), without clinical improvement.

**Figure 2:**
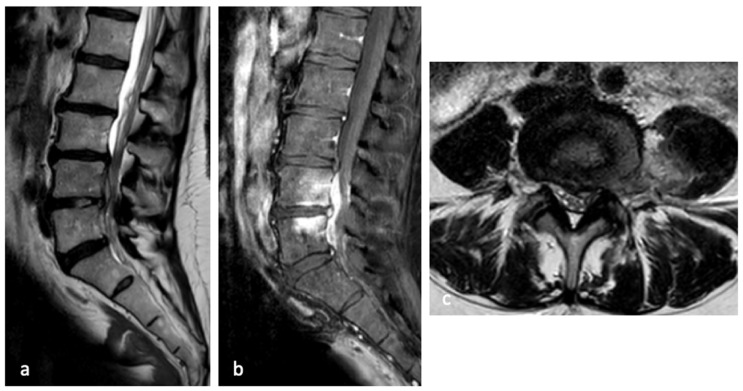
(**a**) T2-weighted sagittal images and (**b**) STIR showing hyperintensity of the L3 and L4 vertebral bodies and the presence of an intracanal abscess with retropulsion of the posterior longitudinal ligament (PLL), without evidence of large disc hyperintensity, as also demonstrated by the axial view (**c**).

After completion of empirical antibiotic therapy and persistence of symptoms, a contrast-enhanced lumbar spine MRI was performed. The imaging demonstrated an increase in the volume of the L4–L5 disc herniation, with more pronounced extension into the left paramedian and intraforaminal zones. Post-contrast sequences revealed abnormal enhancement of the dural sac and vertebral endplates at L4 and L5, suggestive of an infectious process, which had not been observed on the previous MRI performed in February 2022. Additionally, contrast enhancement and edema were noted in the disc, left paravertebral soft tissues, neuroforamen, and the posterior-medial portion of the left psoas muscle (Figure 2).

Based on the radiological and laboratory findings, an infectious etiology consistent with spondylodiscitis was suspected, associated with significant compression of the dural sac and left L4 nerve root.

The patient was referred to our institution in October 2022. On admission, she was alert, afebrile, and hemodynamically stable. General clinical conditions were fair. She presented with severe left-sided lumbosciatalgia in both sitting and standing positions, a positive straight leg raise test, marked gait impairment due to pain, and no neurological deficits. Given the persistence of symptoms, radiological evidence of infection, and neural compression, surgical treatment was indicated. The patient was admitted to our Spine Surgery Unit on 4 November 2022.

On 6 November 2022, she underwent posterior decompression of the spinal canal with drainage of the suspected abscess and biopsy sampling. Intraoperatively, five samples were collected: three tissue specimens for aerobic, anaerobic, and fungal cultures, one for mycobacterial testing, and one for histopathological examination.

Postoperatively, the patient was started on empiric intravenous antibiotic therapy with daptomycin 700 mg once daily and cefepime at 2 g three times daily. Rapid clinical improvement in both back and leg pain was observed, with no new neurological deficits. Mobilization was initiated on postoperative day one.

A custom-made thoracolumbar brace was prescribed and worn continuously for 70 days. Throughout the postoperative course, the patient remained afebrile, and CRP levels progressively decreased, normalizing within 50 days (from 50 mg/dL to 2 mg/dL).

**Figure 3:**
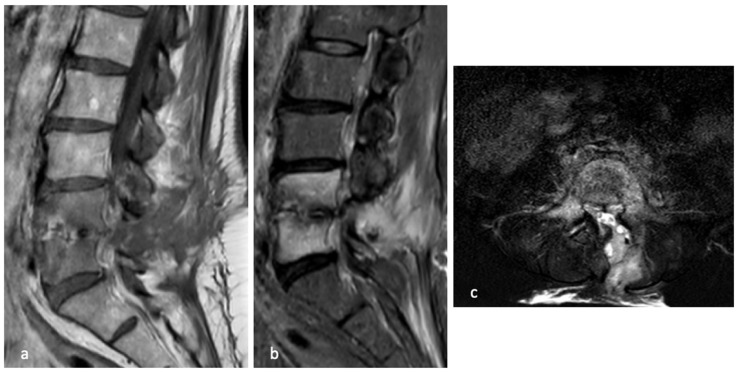
Post-operative MRI in which the surgical approach via and drainage of the intracanal abscess can be seen, in the sagittal T1 (**a**) and T2 (**b**) weighted sequences, as well as signal alteration of the vertebral bodies and disc.

**Figure 4:**
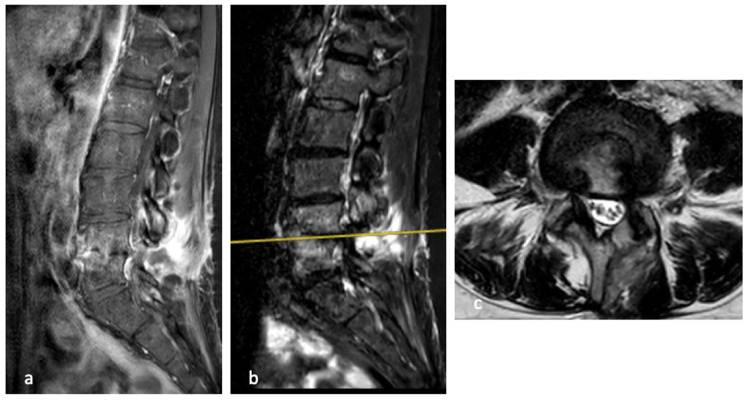
MRI at the resolution of the clinical symptoms and the improvement of the laboratory tests, alterations of the vertebral bodies are still visible in the sagittal T1 (**a**) and T2 (**b**) weighted sequences. In the axial view (**c**), the disc and root are less involved.

In January 2023, a follow-up MRI showed no evidence of active bone infection or residual paravertebral abscesses. Hyperintensity on STIR sequences persisted in the L4 and L5 vertebral bodies, albeit markedly reduced compared to previous imaging. (Figure 3 and Figure 4)

At final follow-up, the patient had discontinued the thoracolumbar brace and reported sustained clinical improvement, with complete resolution of pain and full functional recovery. A chronological summary of the patient’s clinical course, including diagnostic findings, treatments, and outcomes, is provided in Table 1.

## 4. Systematic Literature Review

### 4.1. Demographic and Clinical Presentation

A total of eight published cases of spondylodiscitis following oxygen–ozone therapy were identified in the literature. The patients’ ages ranged from 29 to 67 years, with a predominance of female subjects (six females and two males). Clinical presentation and disease severity varied considerably among the reported cases, reflecting the heterogeneous nature of this complication.

Gazzeri et al. (2007) [7] reported a 57-year-old male who developed spondylodiscitis involving the L4/L5 and L5/S1 levels and who presented with severe sepsis. Despite radiological confirmation by CT and MRI, the patient experienced a rapidly fatal course and died shortly after hospital admission [7]. In contrast, Wu Bo et al. (2009) described a 57-year-old female with extensive cervical involvement from C3 to C7, who presented with quadriplegia, fever, and neck stiffness, associated with an epidural abscess on MRI [8]. Although the neurological presentation was severe, prompt surgical and medical treatment led to complete recovery.

Less dramatic clinical scenarios were reported in other cases. Fort et al. (2014) described a 29-year-old female who presented with low back pain and radicular symptoms related to L5/S1 involvement, without relevant systemic manifestations [9]. Similarly, Salaria et al. (2021) reported a 55-year-old female with L3/L4 spondylodiscitis presenting mainly with localized lumbar pain [10].

Systemic involvement was more evident in the cases described by Cano et al. (2016) and Yang et al. (2018) [11,12]. The former reported a 51-year-old female with cervical spondylodiscitis at C6/C7 complicated by sepsis [11], whereas the latter described a 67-year-old female presenting with urinary retention and an extensive epidural abscess extending from C2 to T1 [12]. Both cases required urgent intervention.

Shahi et al. (2020) presented a 43-year-old female with L4/L5 spondylodiscitis characterized by bilateral radiculopathy and persistent axial pain [13]. More recently, Erroi et al. (2023) reported a 62-year-old male with L3/L4 involvement associated with iliopsoas abscess formation, highlighting the potential for extra-spinal extension of infection [14].

Overall, the anatomical distribution of infection involved the lumbar spine in most cases, followed by cervical localizations, with occasional multilevel involvement. All diagnoses were established by MRI, sometimes complemented by CT imaging.

All demographic characteristics, clinical presentations, and radiological findings of the included cases are summarized in Table 2.

### 4.2. Microbiology and Etiology

Microbiological data were available in most of the reported cases, revealing a heterogeneous spectrum of pathogens, commonly associated with spinal infections, as well as less frequent and opportunistic microorganisms.

Gram-negative organisms were isolated in two cases: Escherichia coli [7] and Achromobacter xylosoxidans [9]. The latter represents an unusual etiology and is typically associated with opportunistic infections, particularly in immunocompromised hosts or in the presence of procedural contamination.

Gram-positive pathogens were identified in three cases: Staphylococcus aureus [8], a well-established cause of vertebral osteomyelitis, Streptococcus beta-haemolyticus [11], and Streptococcus intermedius [12].

Two cases involved a non-tuberculous mycobacterium, Mycobacterium abscessus (Shahi et al.), and *Mycobacterium* spp. [10], underscoring the importance of considering atypical organisms, particularly in subacute or indolent clinical presentations and in cases with delayed diagnosis.

In one report [14], microbiological cultures failed to isolate a specific pathogen, despite suggestive clinical and radiological findings. This emphasizes the diagnostic limitations in certain contexts, especially when empirical antibiotic therapy is initiated prior to sample collection or when fastidious or anaerobic organisms are involved.

### 4.3. Treatment Strategies and Outcome

Gazzeri et al. did not report any surgical or pharmacological intervention [7]. The patient experienced a rapidly fatal course, dying of septic shock shortly after hospital admission. Wu Bo et al. performed posterior cervical decompression (C1–C4) and initiated intravenous antibiotic therapy with ceftazidime and vancomycin [8]. Prompt surgical and medical management resulted in complete neurological and clinical recovery.

Fort et al. treated the infection surgically with anterior lumbar decompression and fusion, followed by antibiotic therapy with vancomycin and ceftriaxone [9]. This combined approach led to a favorable outcome with full functional recovery.

Cano et al. conducted urgent abscess drainage combined with posterior cervical laminectomy and administered vancomycin [11]. The patient responded well to treatment, achieving complete resolution of the infection.

Yang et al. performed surgical drainage of the abscess, followed by antibiotic therapy with ceftriaxone [12]. A favorable clinical and radiological outcome was observed.

Shahi et al. managed the infection with surgical decompression and fusion [13]. Antibiotic therapy was based on clarithromycin, selected according to microbiological sensitivity. Targeted antimicrobial treatment contributed to sustained clinical improvement.

Salaria et al. did not report any surgical or pharmacological intervention [10], with favorable clinical outcomes. Clinical resolution was achieved with conservative management alone. Erroi et al. opted for a conservative, non-surgical approach [14]. The patient received a broad-spectrum antibiotic regimen comprising metronidazole, amoxicillin/clavulanic acid, cephalosporins, and neridronate, resulting in full recovery. This case highlights the potential effectiveness of non-operative management in selected patients without neurological compromise.

In all cases except one, clinical outcomes were favorable, regardless of whether the approach was surgical or conservative. Treatment decisions appeared to be primarily guided by neurological status, systemic involvement, and the presence of abscess formation.

## 5. Discussion

Oxygen–ozone (O_2_–O_3_) therapy is a widely used minimally invasive treatment for discogenic low back pain, particularly in cases of lumbar disc herniation that are unresponsive to conservative measures [15]. The proposed mechanisms of action include disc dehydration and volume reduction, anti-inflammatory effects mediated by inhibition of pro-inflammatory cytokines, and analgesic properties through modulation of pain mediators [16,17]. These effects aim to relieve nerve root compression while preserving spinal stability and avoiding surgical intervention.

Despite its generally favorable safety profile, severe infectious complications have been reported following percutaneous spinal injection procedures, including spondylodiscitis and vertebral osteomyelitis [18,19]. In these cases, pathogenesis is thought to differ from the more common hematogenous spread of infection. Instead, direct inoculation during the injection procedure may lead to localized infection involving the intervertebral disc, vertebral endplates, and adjacent soft tissues [20].

However, in most reported cases, the relationship between O_2_–O_3_ therapy and subsequent infection remains temporal and circumstantial, and a definitive causal link cannot be firmly established. Nevertheless, the absence of alternative sources of infection and the consistent temporal sequence observed in several reports support a plausible association.

In this context, Lactobacillus iners represents a particularly unusual etiologic agent. L. iners is one of the most prevalent species within the human vaginal microbiota [21,22,23]. It contributes to mucosal homeostasis through lactic acid production and inhibition of pathogenic microorganisms [24,25,26]. Interestingly, while Lactobacillus species are traditionally classified as Gram-positive, certain strains of L. iners exhibit variable Gram-staining properties and unique biological characteristics [25].

One of the most distinctive features of L. iners is its ability to produce inerolysin, a pore-forming toxin usually associated with pathogenic bacteria, which may facilitate tissue invasion and immune evasion [27,28]. In addition, its small genome size and specialized metabolic profile suggest a high degree of adaptation to the vaginal environment, potentially enabling opportunistic behavior under specific conditions.

It should be emphasized that most available evidence regarding the biological behavior of L. iners derives from studies on the vaginal microbiome, and extrapolation to spinal infections remains largely hypothetical. Therefore, the pathogenic mechanisms underlying their involvement in spondylodiscitis require further investigation.

To our knowledge, this is the first documented case of spondylodiscitis caused exclusively by L. iners in an immunocompetent patient. The route of infection remains speculative, but contamination during the intradiscal injection procedure followed by local proliferation represents a plausible mechanism.

The findings of our systematic review highlight the marked heterogeneity of microorganisms involved in post-oxygen–ozone spondylodiscitis, ranging from common pathogens such as Staphylococcus aureus and Streptococcus species to Gram-negative bacteria and atypical mycobacteria. One case lacked microbiological confirmation, emphasizing the importance of obtaining adequate tissue samples whenever possible.

Treatment strategies across reported cases varied considerably, including conservative management with antibiotics alone and combined surgical and medical approaches. Clinical outcomes were generally favorable when timely diagnosis and appropriate therapy were instituted, whereas delayed or absent treatment was associated with poor prognosis. Our case further reinforces the central role of early biopsy and microbiological analysis in guiding targeted antimicrobial therapy. Prompt etiological diagnosis, appropriate antibiotic selection, and timely surgical intervention in selected cases remain essential to prevent irreversible neurological damage and systemic complications.

From a preventive perspective, these findings underscore the importance of meticulous aseptic technique, standardized procedural protocols, and careful patient selection when performing minimally invasive spinal injections. Strict adherence to infection-control measures and appropriate operator training are essential to minimize iatrogenic risk.

### Limitations

The main limitation of this study is the rarity of Lactobacillus iners spondylodiscitis, with conclusions necessarily based on a single clinical case and a limited number of published reports. The small sample size and the predominance of case reports and small case series limit the generalizability of our findings and preclude robust statistical analysis. Moreover, the heterogeneity of clinical presentations, diagnostic approaches, and treatment strategies among the included studies further restricts direct comparison. In addition, incomplete microbiological data and variable diagnostic methods in some reports may have influenced the accuracy of pathogen identification. In several cases, prior empirical antibiotic therapy may have reduced culture sensitivity, leading to potential underestimation of certain microorganisms. The retrospective nature of the available literature and the possibility of publication bias, with a tendency to report more severe or unusual cases, represent additional sources of limitation. Finally, the absence of standardized protocols for oxygen–ozone therapy and infection prevention across different centers limits the ability to draw definitive conclusions regarding procedural risk factors.

## 6. Conclusions

Spondylodiscitis due to Lactobacillus iners is an exceptionally rare condition and represents a diagnostic and therapeutic challenge. To our knowledge, this report documents the first confirmed case of this pathogen causing spondylodiscitis following intradiscal oxygen–ozone therapy in an immunocompetent patient. Promising minimally invasive procedures, such as oxygen–ozone infiltrations for lumbar disc disease, despite their favorable safety profile, may be associated with rare but potentially severe infectious complications, particularly when strict aseptic protocols are not rigorously applied.

Given the non-specific clinical presentation and the broad microbiological spectrum, early diagnosis remains challenging. In this context, prompt imaging and surgical biopsy play a pivotal role in confirming the etiology, guiding targeted antimicrobial therapy, and differentiating infectious from non-infectious causes of vertebral pathology. Our experience, together with the findings from the available literature, supports a tailored therapeutic approach combining prolonged, pathogen-directed antibiotic therapy with surgical intervention in selected cases, particularly in the presence of neurological deficits, abscess formation, or spinal instability. Finally, increased awareness among clinicians, strict adherence to standardized procedural protocols, and appropriate operator training are essential to minimize iatrogenic risk and ensure patient safety when performing minimally invasive spinal procedures.

## Figures and Tables

**Table 1:** Chronological timeline of the patient’s clinical course.

Date	Event
January 2022	Onset of left-sided lumbosciatica. Lumbar MRI showed L4–L5 disc herniation.
February 2022	Baseline lumbar MRI without signs of infection.
March 2022	Five sessions of intradiscal oxygen–ozone (O_2_–O_3_) therapy performed.
July 2022	Progressive worsening of low back and sciatic pain.
September 2022	Laboratory tests revealed neutrophilic leukocytosis and CRP 91 mg/L. Empirical ciprofloxacin therapy started.
20–30 September 2022	Ciprofloxacin 500 mg twice daily administered without clinical improvement.
October 2022	Referral to the tertiary spine center. Clinical and radiological suspicion of spondylodiscitis.
4 November 2022	Admission to Spine Surgery Unit.
6 November 2022	Posterior decompression, abscess drainage, and biopsy performed.
November 2022	Empirical intravenous therapy with daptomycin and cefepime initiated.
November 2022–January 2023	Use of thoracolumbar brace for 70 days. Progressive clinical improvement.
January 2023	Follow-up MRI showed resolution of active infection.
Final follow-up	Complete clinical recovery and resolution of pain.

**Table 2:** Demographic data of patients and systematic review.

Author	Year	Age/Gender	Etiology	Level of Infection	Imaging MRI/CT	Clinical Presentation	Surgical Treatment	Antibiotic Treatment	Outcome
Gazzeri et al. [7]	2007	57 M	Escherichia coli	L4/L5 and L5/S1	CT and MRI	Sepsis	-	-	Dead after 3 days due to septic shock
Wu Bo et al. [8]	2009	57 F	Staphylococcus aureus	C3/C7	MRI	Quadriplegia, neck pain, nuchal rigidity and fever with epidural abscess	C1/C4 posterior decompression	Ceftazidime 2 g every 8 h and vancomycin 1 g every 12 h	Full recovery
Fort et al. [9]	2014	29 F	Achromobacter xylosoxidans	L5/S1	CT and MRI	Back and leg pain	Anterior decompression and fusion	Vancomycin and ceftriaxone	Full recovery
Cano et al. [11]	2016	51 F	Streptococcus beta-haemolytic	C6/C7	MRI	Sepsis	Urgent abscess drainage and posterior laminectomy	Vancomycin	Full recovery
Yang et al. [12]	2018	67 F	Streptococcus intermedius	C2/T1	MRI	Extensive SEA, urinary retention	Surgical drainage	Ceftriaxone	Full recovery
Shahi et al. [13]	2020	43 F	Mycobacterium abscessus	L4/L5	MRI	Bilateral radiculopathy and low back pain	Decompression and fusion	Clarithromycin	Full recovery
Salaria et al. [10]; Erroi et al. [14]	2021; 2023	55 F; 62 M	Mycobacterium not isolated	L3/L4	MRI	Low back pain, iliopsoas abscess	-	Metronidazole 500 mg 3 times/day, amoxicillin/clavulanic 3 times/day, cephalosporin; neridronate	Full recovery

## Data Availability

The data supporting the findings of this study are available within the article. Additional details may be available from the corresponding author upon reasonable request. No publicly archived datasets were generated.

## Supplementary Materials

The following supporting information can be downloaded at https://www.mdpi.com/article/10.3390/diseases14030115/s1, Table S1: PRISMA 2020 Checklist [29].
